# Tribulus terrestris for treatment of sexual dysfunction in women: randomized double-blind placebo - controlled study

**DOI:** 10.1186/2008-2231-22-40

**Published:** 2014-04-28

**Authors:** Elham Akhtari, Firoozeh Raisi, Mansoor Keshavarz, Hamed Hosseini, Farnaz Sohrabvand, Soodabeh Bioos, Mohammad Kamalinejad, Ali Ghobadi

**Affiliations:** 1 Department of Traditional Medicine, School of Medicine, Tehran University of Medical Sciences, Tehran, Iran; 2 Psychiatry, Fellow of the European Committee of Sexual Medicine (FECSM), Roozbeh Psychiatric Hospital, Psychiatric and Clinical Psychology, Research Center, Tehran University of Medical Sciences, South Kargar Street, Tehran 13337, Iran; 3 School of Public Health, Tehran University of Medical Sciences, Tehran, Iran; 4 Clinical Trial Center, Tehran University of Medical Sciences, Tehran, Iran; 5 Department of Gynecology and Infertility, Imam Khomeini Hospital, Tehran University of Medical Sciences, Tehran, Iran; 6 Department of Pharmacognosy, School of Pharmacy, Shaheed Beheshti University of Medical Sciences, Tehran, Iran; 7 Department of Traditional Medicine, School of Traditional Pharmacology, Tehran University of Medical Sciences, Tehran, Iran

**Keywords:** Tribulus terrestris, Sexual dysfunction, Women, Traditional medicine

## Abstract

**Background:**

Tribulus terrestris as a herbal remedy has shown beneficial aphrodisiac effects in a number of animal and human experiments. This study was designed as a randomized double-blind placebo-controlled trial to assess the safety and efficacy of Tribulus terrestris in women with hypoactive sexual desire disorder during their fertile years. Sixty seven women with hypoactive sexual desire disorder were randomly assigned to Tribulus terrestris extract (7.5 mg/day) or placebo for 4 weeks. Desire, arousal, lubrication, orgasm, satisfaction, and pain were measured at baseline and after 4 weeks after the end of the treatment by using the Female Sexual Function Index (FSFI). Two groups were compared by repeated measurement ANOVA test.

**Results:**

Thirty women in placebo group and thirty women in drug group completed the study. At the end of the fourth week, patients in the Tribulus terrestris group had experienced significant improvement in their total FSFI (p < 0.001), desire (p < 0.001), arousal (p = 0.037), lubrication (p < 0.001), satisfaction (p < 0.001) and pain (p = 0.041) domains of FSFI. Frequency of side effects was similar between the two groups.

**Conclusions:**

Tribulus terrestris may safely and effectively improve desire in women with hypoactive sexual desire disorder. Further investigation of Tribulus terrestris in women is warranted.

## Background

Hypoactive sexual desire disorder (HSDD) is a multi factorial and multifaceted disorder which has been entangled with biologic and psychological considerations and interpersonal relations. HSDD is a common sexual complaint affecting approximately 1 in 10 adult women in the USA [1,2], and its prevalence appears to be similar in Europe (7%-16%) [3] and Australia (16%) [4]. According to some scientific reports the prevalence in Iran is 30% [5].

Currently there are two therapeutic modalities for women who suffer from the lack of libido: psychotherapy and pharmacotherapy. Several drugs have been suggested for treating women’s loss of libido including estrogen, methyl testosterone [4], phenethylamin [5], bupropion [6] and saffron [7]. However, there is no Food and Drug Administration (FDA) approved drug for treating women’s HSDD [8,9].

Avicenna’s *The Canon of Medicine* (1025 AD) provides a full chapter to the description of libido, its disorders and treatments. Twentieth chapter of the book discusses the libido under rubric of “*Bah”*, its disorders and causes that may boost and reduce the sex drive as well as treatments for each cause [10]. According to *the Canon of Medicine* and Aghili Khorasani’s *Makhzan al-Advia* (The treasury of Spices) (18th AD), Bindii or *Tribulus terrestris* influences libido [11] and is able to boost sex drive in human beings. According to the Iranian Traditional Medicine (ITM) deregulation and disorders of libido in both sexes is considerably common. It is worthy to mention that, ITM is based on principles of Humorist school and four humors (black bile, yellow bile, phlegm and blood), but the mentioned herbal medicine, Tribulus terrestris, has been classified as an experimental remedy because it affects all four humors and boosts the libido.

Recent scientific studies on medicinal herbs have referred to Tribulus terrestris as an effective drug of women’s sex drive [12]. A study on mice hypothesized that the drug may affect follicle-stimulating hormone (FSH) and luteinizing hormone (LH); injecting 10 mg of Tribulus extract into female mice resulted in significant increase in both growth rate and size of follicles in comparison to the control mice [13].

The aim of our clinical trial was to analyze the effect of Tribulus on women with HSDD in childbearing age.

## Methods

### Trial design

This was a randomized, double-blind, placebo-controlled clinical trial study, performed in two medical centers in Tehran Province, Rostamabad Neighborhood Health Center, part of the 1st district of the greater Tehran Municipality, and Sajjad Hospital in city of Shahryar. This study was approved by the ethics committee of School of Medicine, Tehran University of Medical Sciences (TUMS), and it was registered in the clinical trial center under reg. no. of IRCT20121111111425N1

### Participants

Participants of the study were selected after responding to this specific question “*Do you suffer from loss of libido?”* in a direct and face to face encounter, and the study was conducted from June 2012 to July 2013. Then a phone interview was scheduled for all 96 women who were selected; and subsequently they were invited for a screening visit after verification of their disorder. The inclusion criteria of the study were: being married and actively living with a partner at least 15 days per month, being in childbearing age, lack and or loss of libido which causes distress, having normal pelvic exam, negative pap smear test conducted at least within past six months, and having normal breast exam. The exclusion criteria were: lack of steady sexual partner, suffering from a serious medical condition (any disease which may force the participant to use drugs during the study), any history of genital tract or breast cancers, suffering from major depression disorder or other psychiatric disorders, menopause, pregnancy, husband’s sexual problems and active plans for divorce. Participants with the mentioned inclusion criteria were allowed to start the next stage which was a face to face interview based on Diagnostic and Statistical Manual of Mental Disorders (DSM-IV-TR) codes for HSDD [14]. Finally patients were asked to sign the informed consent forms.

### Preparations of tribulus terrestris

Tribulus is widely scattered across the planet a herbal plant [15]. Tribulus belongs to Zygophyllaceae family. Alpha amyrin constitutes over 60% of active ingredient (AI) of the herb [16]. Dried leaves of Tribulus were obtained from a licensed distributer of herbs and natural remedies in Tehran. They were approved and registered by the SHUM herbarium (voucher number: SHUM 8051).

### Preparations of syrup and placebo

Traditional Medicine Department, School of Medicine, TUMS used the whole vaporized ethanolic extract of Tribulus terrestris for preparing the syrup. There was 3.5 grams of ethanolic extract in every 5 ml of the syrup. The placebo was prepared via the same method without Tribulus terrestris extract as a sucrose syrup.

### Treatment and assessment

Each participant was initially asked to fill the Female Sexual Function Index (FSFI) (21) a self-reported questionnaire under supervision of an Iranian traditional medicine intern who was already trained by a gynecologist and a psychiatrist for conducting the trial, and then she was provided with a drug bottle with specific cod*ing*. Drug Product Information Form (DPIF) was inscribed on a label attached to the bottle. The specific code was recorded on both questionnaire and participant’s data sheet. The DPIF instructions included this information: 7.5 ml of the drug should be used two times a day for four full weeks since the day after the completion of menstruation period. Two days after submitting the drug, the patient was checked by a physician through a phone call in order to make sure that the drug was used correctly. Again, after a week a second phone call was made to make sure of continuity of the mentioned order. Patients, who forgot to use more than three doses of their drugs, were excluded. Another interview according to the DSM-IV-TR criteria for HSDD was made four weeks after completion of the drug therapy, side effects (if any) were checked and FSFI questionnaire was refilled. The participants were allowed to call their therapist during the treatment period.

### Outcomes

The participants filled the FSFI questionnaire [1,17] two times: 1) at the beginning of the study, 2) four weeks after finishing their drug. The questionnaire have 19 questions with six domains included desire, arousal, lubrication, orgasm, satisfaction and pain, and a general score in which higher scores reflected better sexual activity. Questions 1 and 2 were related to the sexual desire and its score varied between 1.2 and 6. Questions 3, 4, 5 and 6 analyzed arousal of the patient. Another domain of the questionnaire was lubrication which covered questions 7, 8, 9, and 10. The orgasm domain included questions 11, 12 and 13. Questions 14, 15 and 16 all were related to the sexual satisfaction and finally questions 17, 18 and 19 analyzed pain.

The initial outcome was the assessment of desire and total score of our samples with FSFI before and after intervention. Likewise, different subscales including arousal, lubrication, orgasm, satisfaction and pain were compared as the secondary targets using the questionnaire before and after treatment. The FSFI question contained 19 questions which analyzed five domains of women’s sexual function. Side effects were recorded systematically within the trial period through visits made within 1, 2 and 4 weeks and after the end of pharmacotherapy.

### Sample size

The statistical power was set at 80% in order to achieve a minimum score of 3, to improve the “sexual desire” score in the intervention (experimental) group in comparison with the control group. The standard deviation (SD) was 3.5 (resulted from our pilot study). In regards to possibility of loss measuring 20% during the study period, we achieved significance level of 95% and a bilateral *t*-test.

### Randomization, allocation concealment, and blinding

The randomized sequence was produced by a methodology using randomization permuted block technique with blocks size of 4 which was submitted to therapists with ratio 1:1 divided in two groups. The randomized sequence for allocating the main or placebo treatment was concealed through producing unknown codes; hence, pre-provided drugs and placebo were submitted through similar packs but different codes, as neither therapists nor patients were aware of the active ingredient of the drug packs.

### Statistical analysis

Descriptive baseline characteristics for two groups comparisons were tabulated as means and SD or as percentages. All analyses comparing the efficacy of our primary and secondary outcomes were by intention-to-treat principles. Using General Linear Model (GLM) score of desire, arousal, lubrication, orgasm, satisfaction, and pain between two groups were compared by repeated measurement ANOVA test. Compound symmetry assumption was tested using Mauchley’s Sphericity test. The time groups cross-product (interaction term) was considered as group differences in their response over time with the baseline values as a covariate in this model. Significance level of 5% (alpha = 0.05) was used for all statistical tests.

## Results

**Figure 1:**
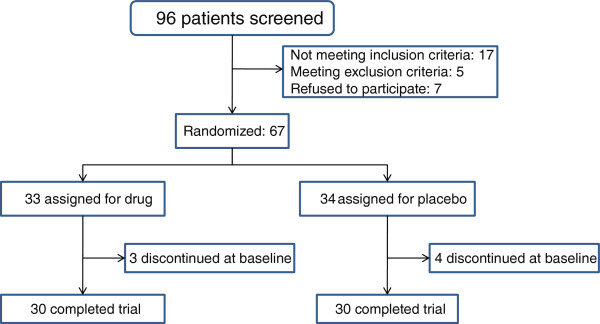
Flow chart indicating subject enrolment, group allocation and analysis according to CONSORT guidelines.

A total of 96 women were enrolled for the study (Figure 1), and 67 women were identified as qualified (out which two women decided not to participate after acquiring DSM IV codes and upon issuing the informed consent, three other women stopped participating at the second phone call time point despite receiving drug and answering the first phone call. According to the code recorded on the questionnaire, (before taking drug) it was turned out that two women were from the placebo group and another one was from the drug group. Their reasons for stopping participating at the studying included very sweet taste of the drug (one woman) and anxiety due to using an investigational drug (two women). Another two women were not available after getting off the study; they signed the consent form, but there was no drug available at that time when they were in the center, and they never came back to receive their ration. Between June 2012 and July 2013, participants took part in an interview and then they filled the FSFI questionnaire; then they were assigned randomly in drug and placebo groups. There was a second visit a week after starting pharmacotherapy and third visit was made four weeks after termination of the treatment which was accompanied with the filling of the questionnaire for the second time. Hence patients in average were followed up for two consecutive months (Figure 1).

60 women out of 67 participants completed their treatment process and the results were analyzed according to what was recorded in their questionnaires. The mean ages of drug and control groups were 36 ± 6.24 years and 36.13 ± 5.88 years, respectively. 70% and 66.6% of participants in drug and control groups had bachelor or higher educational degrees (Table 1). 30 members of Tribulus and 30 members of the placebo group completed their treatment process.

**Table 1:** Demographic and baseline characteristics of patients

		**Tribulus (n = 30)**	**Placebo (n = 30)**
Age (years), mean ± SD		36 ± 6.24	36.13 ± 5.88
Partner age, mean ± SD		39.87 ± 7.13	40.43 ± 7.74
BMI kg/m^2^		26.25 ± 3.75	24.67 ± 2.92
Education	Diploma	9	10
	Bachelor	21	17
	MA	0	3
Delivery (mean ± SD)			
Type of delivery	No	10%	6.7%
	NVD	60%	40%
	C/S	30%	53.3%
Contraception	Natural	33.3%	43.3%
	OCP	10%	6.7%
	Candom	23.3%	13.3%
	IUD	16.7%	13.3%
	None	10%	3.3%
	Tubectomy	6.7%	20%
Marriage years		14 ± 5.97	12.73 ± 6.22
mean ± SD			

Patients’ desire was the main measured outcome of the study in both groups (Table 2) which was measured after four weeks of pharmacotherapy process by using the Female Sexual Function Index (FSFI) questionnaire and the following scores were gained: score of placebo group, before and after intervention, respectively: 2.66 ± 0.75 and 2.86 ± 0.79; scores of Tribulus group, before and after intervention, respectively: 3.06 ± 0.69 and 3.90 ± 0.71. After matching the effect of the possible effective variables (age, pregnancy rate, method of delivery, method of contraception), the difference became significant statistically (*p* < 0.001).

**Table 2:** Summary results for each study group

			**Mean differences**	**Placebo**	**Tribulus terrestris**
			**(CI 95%)**	**N = 30**	**N = 30**
	**Control**	**Baseline**	**Control**	**Baseline**	
	**Mean ± SD**	**Mean ± SD**	**mean ± SD**	**mean ± SD**	
Desire	3.66 ± 0.69	3.90 ± 0.71	2.66 ± 0.75	2.86 ± 0.79	0.71 (0.41 – 1.01) p < 0.001
Arousal	3.61 ± 0.92	4.21 ± 0.67	3.16 ± 0.95	3.17 ± 0.75	0.75 (0.47 – 1.46) p = 0.037
Lubrication	4.15 ± 1.13	4.66 ± 0.87	4.15 ± 1.15	4.18 ± 0.79	0.50 (0.32 – 0.68) p < 0.001
Orgasm	3.21 ± 0.98	4.20 ± 0.72	3.31 ± 0.97	3.59 ± 0.85	0.85 (0.56 – 1.14) p < 0.001
Satisfaction	3.44 ± 1.15	4.61 ± 0.93	3.43 ± 1.13	3.75 ± 1.12	1.10 (0.73– 1.48) p < 0.001
Pain	4.19 ± 1.56	5.07 ± 1.01	4.19 ± 1.51	4.87 ± 1.42	0.37 (0.16 – 0.73) p < 0.001
General score	22.41 ± 2.87	26.80 ± 3.03	20.39 ± 4.64	21.25 ± 4.72	4.32 (3.33 – 5.31) p = 0.040

Another variable analyzed in this study was arousal which was measured four weeks after completion of the treatment process through the FSFI questionnaire. Scores of placebo group before and after pharmacotherapy were 3.16 ± 0.95 and 3.17 ± 0.75; Scores of intervention group before and after using Tribulus terrestris were 3.61 ± 0.92 and 4.21 ± 0.67. After matching the effect of the possible effective variables (age, pregnancy rate, method of delivery, method of contraception), the difference became significant statistically (*p* = 0.037).

According to the FSFI another secondary variable was lubrication. Similar to two previous variables lubrication was analyzed four weeks after completion of the treatment. Scores of this domain gained by placebo group before and after usage were 4.15 ± 1.15 and 4.18 ± 0.79 while scores won by intervention group were 4.15 ± 1.13 and 4.66 ± 0.87. After matching the effect of the possible effective variables (age, pregnancy rate, method of delivery, method of contraception), the difference became significant statistically (*p* < 0.001).

Orgasm was analyzed using the FSFI questionnaire after four weeks of completion of treatment with Tribulus terrestris. Scores of this domain gained by placebo group before and after usage were 3.31 ± 0.97 and 3.59 ± 0.85 while scores won by intervention group were 3.21 ± 0.98 and 4.20 ± 0.72. After matching the effect of the possible effective variables (age, pregnancy rate, method of delivery, method of contraception), the difference became significant statistically (*p* < 0.001).

Satisfaction was measured four weeks after completion of treatment process through the FSFI questionnaire. Scores of this domain gained by placebo group before and after pharmacotherapy were 3.43 ± 1.13 and 3.75 ± 1.12; scores won by intervention group before and after using Tribulus terrestris were 3.44 ± 1.15 and 4.61 ± 0.93. After matching the effect of the possible effective variables (age, pregnancy rate, method of delivery, method of contraception), the difference became significant statistically (*p* < 0.001).

Pain was measured four weeks after completion of treatment process through the FSFI questionnaire. Scores of this domain gained by placebo group before and after pharmacotherapy were 4.19 ± 1.51 and 4.87 ± 1.42; scores won by intervention group before and after using Tribulus terrestris were 4.19 ± 1.56 and 5.07 ± 1.01. After matching the effect of the possible effective variables (age, pregnancy rate, method of delivery, method of contraception), the difference became significant statistically (*p* = 0.041).

Finally general scores of both drug and placebo groups were analyzed and compared based on FSFI questionnaire four weeks after completion of intervention. The general scores in placebo and intervention groups before using Tribulus terrestris were 20.89 ± 6.46 and 21.25 ± 4.72, respectively; and four weeks after its completion were 22.41 ± 2.87 and 26.80 ± 3.03, respectively (p = 0.040). These results confirmed by the interviews using DSM-IV-TR (Table 2).

### Safety assessment

Possible side effects including abdominal pain, cramping, nausea, vomiting, diarrhea or constipation were recorded using Common Terminology Criteria for Adverse Events (CTCAE) Version 4.02 during the scheduled visits. Only one patient reported grade 1 abdominal cramp.

## Discussion

Our findings showed that Tribulus terrestris was effective in improving women’s sex drive based on the questions of FSFI questionnaire. Since the basic findings of two groups were similar, the improved score of the Tribulus group can be attributed to libido boosting effects of the plant. Our findings confirmed what has already been described in the Iranian Traditional Medical textbooks about libido boosting effects of Tribulus. There is also more recent evidence that Tribulus can be used as a libido booster plant in women [12]. There may be a possible synergy between Tribulus and FSH-LH mechanism. For instance, one animal experiment showed that mice, injected with 10 mg Tribulus extract, developed thicker theca internal layer and more follicles in comparison with control mice in the same period of time [13].

Prescribing Tribulus extract for women can increase desire score in women who suffer from loss of sex desire. However, according to our knowledge there has not been any similar research on the impact of Tribulus extract in improving women’s sex desire in childbearing age. The effect of testosterone in improving desire in menopausal women has been confirmed by studies that have looked at women’s sex drive and how to boost it [18,19]. The role of conjugated testosterone in treating lack of sex drive has been emphasized [20]. Currently testosterone is the only approved drug for treatment of HSDD in menopausal women, particularly for those have surgically-induced menopause [21]. In one study on women with SSRI induced desire disorder, it was shown that Saffron was an effective herbal remedy which could improve desire [7]. Another study showed that Bupropion was effective in improving women’s desire [6]. However, Tribulus is the only drug which not only improves the sexual desire in women but also it does not have any unexpected side effects in patients.

Our study showed very clearly through acquired scores of the FSFI questionnaire that patients had significant increase in their scores related to questions about desire, arousal and satisfaction. Our results indicated a considerable improvement in scores of desire. Thus, Tribulus had apparent boosting effects on desire, and it was able to improve sexual satisfaction and sexual behavior.

Although our study showed the effect of Tribulus on improving the desire, it must be noticed that Iranian women are generally modest and shy because of their cultural beliefs about sexual concepts, and our sample size was small. In order to produce consistent results and more reliable evidence, more studies with a large sample size must be carried out.

## Conclusion

Tribulus terrestris may safely and effectively improve desire in women with HSDD. Further investigation of Tribulus terrestris in women is warranted.

## Competing interests

The authors do not have any financial/commercial competing interest in the study presented here. This study was supported with Tehran University of Medical Sciences, School of Traditional Medicine.

## Authors’ contribution

EA was responsible for recruiting, visiting and evaluating patients; she was also involved in designing the study, analyzing the data and writing the manuscript. FR (the corresponding author) supervised the trial and was involved in all aspects of the study including designing and coordinating the study as well as analyzing the data and drafting the manuscript. MK gave his valuable comments throughout the study and was involved in the original design of the proposal, and also the final revision of the manuscript. HH performed all statistical analysis of the data and interpreted the results of the study. FS was the consultant gynecologist who recommended and supervised the gynecological evaluations of the patients. SB was consulted for a list of medications or herbal products that could interfere with the results of this study. MK participated in the identification of the plants, plant extraction and supervised preparing the placebo and Tribulus terrestris extract. AG prepared the placebo and Tribulus terrestris extract under supervision of MK. All authors read and approved the final manuscript.
